# Spontaneous lung pathology in a captive common marmoset colony (*Callithrix jacchus*)

**DOI:** 10.5194/pb-4-17-2017

**Published:** 2017-03-01

**Authors:** Martina Bleyer, Marius Kunze, Eva Gruber-Dujardin, Kerstin Mätz-Rensing

**Affiliations:** Pathology Unit, German Primate Center, Kellnerweg 4, 37077 Göttingen, Germany

## Abstract

Data on spontaneous pathology are substantially scarce for common marmosets, compared to other laboratory animals, but is essential for the interpretation of histological findings in the context of toxicological and experimental studies. Especially if common marmosets are used as experimental animals in respiratory research, detailed knowledge on the spectrum, occurrence, and incidence of spontaneous histopathological pulmonary lesions in this non-human primate species is required. In this study, lung tissue of 638 common marmosets from the marmoset colony of the German Primate Center was examined histologically. The analysis revealed a high incidence of predominantly mild and multifocal interstitial pneumonia (32.99 %) of unknown etiology in most cases. Only few marmosets exhibited lobar pneumonia (1.41 %) and bronchopneumonia (0.94), which were mainly caused by bacterial pathogens such as *Bordetella bronchiseptica* and *Klebsiella pneumoniae*. Lung immaturity and atelectasis were common histological findings in newborn marmosets. Typical background lesions included anthracosis (8.15 %), hemosiderosis (1.72 %), extramedullary hematopoiesis (11.6 %), mineralization (10.97 %), and inflammatory cell foci (10.34 %). In addition, three cases of pulmonary arteriopathy (0.47 %) and 1 case of foreign-body granuloma (0.16 %) were detected in the marmoset study cohort. The high prevalence of circulatory disturbances (congestion, edema, hemorrhage) and changes in air content (secondary atelectasis, alveolar emphysema) could partly be explained by euthanasia-related artifacts or agonal changes. The present study provides a comprehensive overview of the range and incidence of spontaneous pulmonary histopathology in common marmosets, serving as valuable reference data for the interpretation of lung lesions in toxicological and experimental marmoset studies.

## 1 Introduction

Recently, the common marmoset (*Callithrix jacchus*) has increasingly attracted attention as a translational animal model in the field of respiratory research because of its small size, good availability, and consistent characteristics of primate lung architecture (Greenough et al., 2005; Lever et al., 2008; Seehase et al., 2012; Curths et al., 2013, 2014). As a non-rodent species, the common marmoset is used in preclinical testing of drugs acting on the respiratory system and is a suitable animal model for various human pulmonary diseases, including asthma and chronic obstructive pulmonary disease (COPD; Seehase et al., 2012; Curths et al., 2013, 2014). Histopathological examination of lung tissue from toxicological and experimental studies requires detailed knowledge of the spectrum of spontaneously occurring lung pathology of this laboratory animal species to identify possible drug-induced or disease-associated pulmonary lesions and to distinguish these from species-specific background lesions. Compared to other laboratory animals, spontaneous pathology of common marmosets is less well defined. Background lesions of the common marmoset in toxicological studies have previously been documented by Kaspareit et al. (2006), who also referred to pulmonary findings. David et al. (2009) also performed a retrospective study on the spontaneous pathology of common marmosets including the morphological diagnoses of pneumonia, atelectasis, pulmonary extramedullary hematopoiesis, and lymphosarcoma in the lungs. However, a detailed survey about the range and incidence of lung pathology in common marmosets does not exist in the literature so far.

In order to provide reference data on spontaneous histopathological pulmonary findings in conventionally kept common marmosets, we performed a retrospective study on necropsy material of 638 common marmosets from the indoor-housed marmoset colony of the German Primate Center in Göttingen.

## 2 Materials and methods

In this retrospective study, archived lung tissue of 638 common marmosets (317 males and 321 females) originating from the marmoset colony of the German Primate Center in Göttingen, Germany, was used. Archived material included formalin-fixed or paraffin-embedded lung tissue, or histological sections of the lung, which were collected between 1997 and 2011. Animals of this study were housed in small family groups in an indoor facility with a room temperature of 25 ${}^{\circ }$C and relative humidity of 50–60 % on a 12 h light–dark cycle with 30 min “dawn” and “dusk” periods. Care and housing conditions of the animals complied with the regulations of the European Parliament and the Council Directive on the protection of animals used for scientific purposes (2010/63/EU), the National Institutes of Health Guide for the Care and Use of Laboratory Animals (2010), and the applicable German Animal Protection Law. According to necropsy records, animals underwent necropsy after spontaneous death, following euthanasia due to illness with poor prognosis, or after scheduled terminal kill in the context of experimental studies. From the latter group of marmosets only control animals or animals without treatment-related findings were considered for re-evaluation of lung histology. Photographic documentation of respective macroscopic pulmonary findings and results from bacteriological culture of the lungs were available for some animals. Lung tissue samples were fixed in 4 or 10 % phosphate-buffered formaldehyde, paraffin-embedded, sectioned at 3 µm, and stained with hematoxylin and eosin (HE). If required for diagnostic purposes, additional stains were prepared and analyzed, including periodic acid Schiff (PAS) reaction, Prussian blue stain, von Kossa stain, Masson's trichrome stain, Congo red stain, Grocott's methenamine silver stain, and Giemsa stain. The lungs of all 638 marmosets were re-examined histologically, and findings were reported on a spreadsheet (Microsoft Office Excel 2010) with searchable columns for morphological diagnosis/histological finding, sex, age, cause of death (if known), chronicity of lesion (in case of inflammation), and severity grade. Animals were assigned to three age groups: newborn (0 to 7 days old, including supposedly timely delivered but stillborn marmosets), juvenile (7 days to 30 months old), and adult (older than 30 months). Histological findings were grouped into inflammatory conditions, neoplasia, changes in air content, circulatory disturbances, pigment deposition, and miscellaneous findings. Total incidences of findings were indicated in absolute numbers and percentage. In addition, absolute numbers of findings were calculated for males and females as well as different age groups.

**Figure 1:**
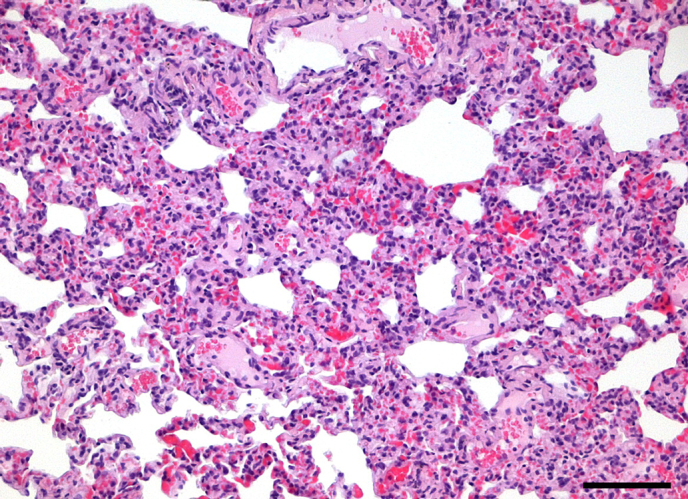
Mild subacute multifocal to coalescing interstitial pneumonia in an adult male common marmoset. Alveolar septa are thickened by infiltrates of lymphocytes, plasma cells, macrophages, and few neutrophils. There is concurrent congestion and evidence of minimal multifocal hemorrhages. HE, scale bar = 100 µm.

## 3 Results

In the present study, 39 of 638 common marmosets (6.11 %) did not show any histological changes of the lungs. All spontaneous pulmonary lesions of the other animals are documented in Table 1.

**Table 1:** Histological findings in the lungs of common marmosets and their corresponding incidences in males and females.

Morphological diagnoses			Total		%		Male		Female		Newborn		Juvenile		Adult	
			(n=638)				(n=317)		(n=321)		(n=133)		(n=176)		(n=328)	
*Inflammation*																
	Interstitial pneumonia		206		32.29		101		105		33		79		94	
		acute		32		5.02		11		21		3		13		16
		subacute		132		20.69		68		64		26		50		56
		chronic		42		6.58		22		20		3		16		23
		with alveolitis		2		0.31		1		1		0		1		1
	Lobar pneumonia		9		1.41		3		6		1		3		5	
		purulent		5		0.78		2		3		1		2		2
		fibrinopurulent		2		0.31		1		1		0		1		1
		with pleuritis		2		0.31		0		2		0		1		1
	Bronchopneumonia		6		0.94		2		4		1		3		2	
	Bronchointerstitial pneumonia		2		0.31		1		1		0		1		1	
*Neoplasia*																
	Lymphoma		4		0.63		2		2		0		1		3	
	Fibrosarcoma		1		0.16		0		1		0		1		0	
*Changes in air content*																
	Atelectasis		215		33.7		95		120		60		49		106	
		primary (fetal)		37		5.8		21		16		36		1		0
		secondary		178		27.9		74		104		24		48		106
	Alveolar emphysema		154		24.14		76		78		11		41		102	
*Circulatory disturbances*																
	Congestion		265		41.54		135		130		37		81		147	
	Edema		131		20.53		64		67		29		40		62	
		alveolar		120		18.81		57		63		26		35		59
		interstitial		2		0.31		1		1		0		0		2
		alveolar and interstitial		9		1.41		6		3		3		5		1
	Hemorrhage		39		6.11		19		20		4		15		20	
	Hyaline membranes		3		0.47		1		2		3		0		0	
*Pigment deposition*																
	Anthracosis		52		8.15		22		30		3		8		41	
	Hemosiderosis		11		1.72		5		6		0		2		9	
*Miscellaneous*																
	Extramedullary hematopoiesis		74		11.6		30		44		2		12		60	
	Mineralization		70		10.97		27		43		0		13		57	
		interstitial		42		6.58		17		25		0		7		35
		subpleural		28		4.39		10		18		0		6		22
	Inflammatory cell foci		66		10.34		32		34		18		17		31	
	Lung immaturity		62		9.72		34		28		61		1		0	
	Alveolar histiocytosis		26		4.08		13		13		2		10		14	
	Fibrosis		23		3.61		10		13		0		8		15	
		interstitial		16		2.51		9		7		0		8		8
		subpleural		7		1.1		1		6		0		0		7
	Arteriopathy		3		0.47		2		1		0		0		3	
	Foreign-body granuloma		1		0.16		0		1		0		0		1	
*None*			39		6.11		175		165		5		10		24	

**Figure 2:**
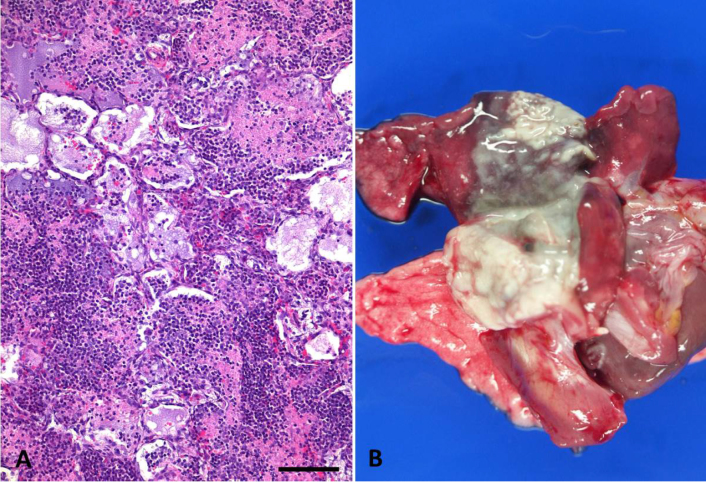
**(a, b)** Severe diffuse acute to subacute fibrinopurulent lobar pneumonia in a female juvenile common marmoset. Alveoli are filled with degenerate neutrophils, macrophages, fibrin, edema fluid, and necrotic debris. HE, scale bar = 100 µm **(a)**. Grossly, there is abundant suppurative exudate on the cut surface of the affected lung lobes **(b)**. In this specific case, bacterial culture was negative.

The most commonly observed inflammatory lung condition was constituted by interstitial pneumonia, which was observed in 206 marmosets (32.29 %). The majority of cases with interstitial pneumonia revealed a subacute course of disease with predominance of plasma cells in the inflammatory cell infiltrate. Regarding severity and distributional pattern, mild multifocal or multifocal to coalescing forms predominated (Fig. 1), while severe and diffuse cases were very rare. In two marmosets (0.31 %), interstitial pneumonia was associated with acute to subacute alveolitis. There was no histological evidence of infectious agents in all cases of interstitial pneumonia, except for one male juvenile marmoset, which showed characteristic disseminated Grocott-positive blastospores and pseudohyphae in inflamed lung regions, indicating a mycotic etiology consistent with candidiasis. Bacterial culture, if available, was positive in the minority of marmosets affected by interstitial pneumonia. Bacterial isolates included *Escherichia coli*, *Streptococcus* sp., *Erysipelothrix rhusiopathiae*, *Klebsiella pneumoniae*, and *Pseudomonas aeruginosa*. Other forms of pneumonia were rare and included lobar pneumonia in nine marmosets (1.41 %), suppurative bronchopneumonia in six marmosets (0.94 %), and bronchointerstitial pneumonia in two marmosets (0.31 %). Lobar pneumonias were further subdivided into purulent or fibrinopurulent forms according to the inflammatory exudate (Fig. 2). In two cases (0.31 %), there was fibrinopurulent pleuropneumonia. The majority of lobar pneumonias (eight of nine cases) and all suppurative bronchopneumonias were moderate to severe and acute to subacute, representing the main cause of disease or death in most cases. Bacterial culture of marmoset lungs affected by purulent bronchopneumonia yielded isolates of *Streptococcus* sp. and/or *Bordetella bronchiseptica* in all cases. *Bordetella bronchiseptica* was also isolated from the lungs of a juvenile female marmoset with fibrinopurulent pleuropneumonia. In three cases of lobar pneumonia, *Streptococcus* sp., *Enterococcus* sp., and/or *Klebsiella pneumoniae* ssp. *ozaenae* could be isolated, while five cases of lobar pneumonia were negative for bacterial culture.

**Figure 3:**
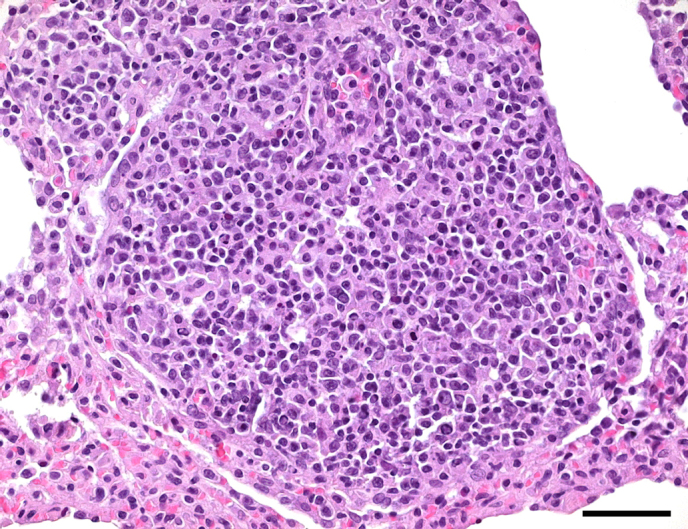
Pulmonary lymphoma in a female juvenile common marmoset. Pleomorphic lymphoblasts with considerable mitotic activity infiltrate the lung parenchyma. HE, scale bar = 50 µm.

Neoplastic conditions occurred in five marmoset lungs, including lymphoma in four adult animals (0.63 %) and fibrosarcoma in one juvenile animal (0.16 %). Regarding the age of animals with tumors, lymphomas affected three rather young adults (3.5 years (two cases) and 2.75 years old) and one older animal (7 years old). The fibrosarcoma occurred in a 1-year-old marmoset. In all cases, pulmonary tumors were regarded as secondary, resulting from metastatic neoplastic disease with presumptive primary tumors in the nodal or extranodal lymphatic system (lymphomas) and in the mammary gland (fibrosarcoma). Immunohistochemical examinations confirmed B cell origin of at least three lymphomas (Fig. 3). One lymphoma has not been further characterized.

Changes in air content were commonly observed, either in otherwise healthy lungs or as an additional finding to other histological diagnoses. There was evidence of atelectasis in 215 marmosets (33.7 %), of which the majority represented subtotal secondary (acquired) atelectasis (178 of 215 cases). Primary (fetal) atelectasis occurred in 37 newborn marmosets (5.8 %), of which 36 animals showed total fetal atelectasis that was regularly associated with lung immaturity. One juvenile marmoset revealed partial fetal atelectasis, also accompanied by discrete signs of lung immaturity. Alveolar emphysema of variable severity and extent was present in 154 animals (24.14 %), whereas interstitial emphysema could not be observed in this study.

Circulatory disturbances in marmoset lungs included congestion, edema, hemorrhage, and hyaline membrane formation. Acute pulmonary congestion was a common finding (41.54 %), often regarded as agonal or euthanasia-induced due to the use of barbiturates. The same might be true for pulmonary edema, which was present in 131 marmosets (20.35 %) and occurred both as an additional finding and solitarily. The majority of pulmonary edema was represented by alveolar forms (120 of 131 cases), while involvement of the interstitium was only seen in 11 cases. Extravasation of erythrocytes (hemorrhage) into the interstitium or alveolar space could be seen in 39 marmosets (6.11 %) and, to some extent, was presumably also caused by euthanasia or agony. Hyaline membranes were observed in the lungs of three newborn marmosets (0.47 %) with concurrent atelectasis and evidence of lung immaturity.

**Figure 4:**
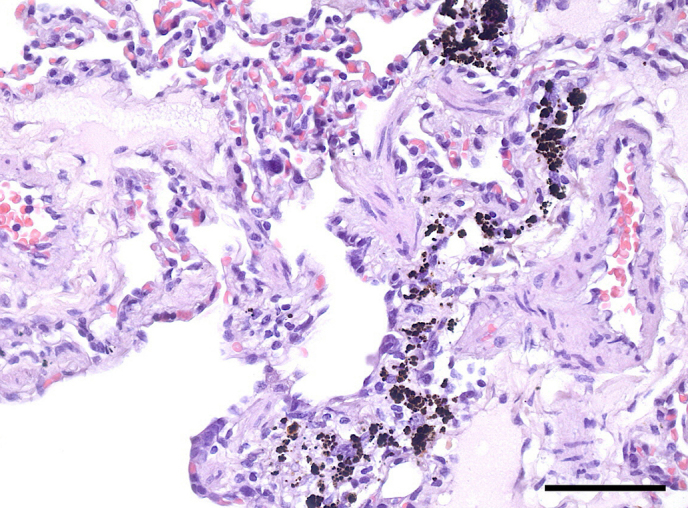
Pulmonary anthracosis in an adult male common marmoset. Dark-brown-to-black pigment is located in perivascular and peribronchiolar areas. There is mild concurrent pulmonary congestion. HE, scale bar = 50 µm.

Mild to moderate deposition of coal dust in the pulmonary interstitium (anthracosis) was present in 52 mostly adult marmosets (8.15 %). In general, anthracosis was not associated with any tissue reaction (Fig. 4). Hemosiderin-laden macrophages (hemosiderosis) were observed in the lungs of two juvenile and nine adult marmosets (1.72 %), which commonly showed co-existing hemosiderosis in other organs, especially in liver, spleen, and kidneys. There was no evidence of chronic heart failure in cases of pulmonary hemosiderosis.

**Figure 5:**
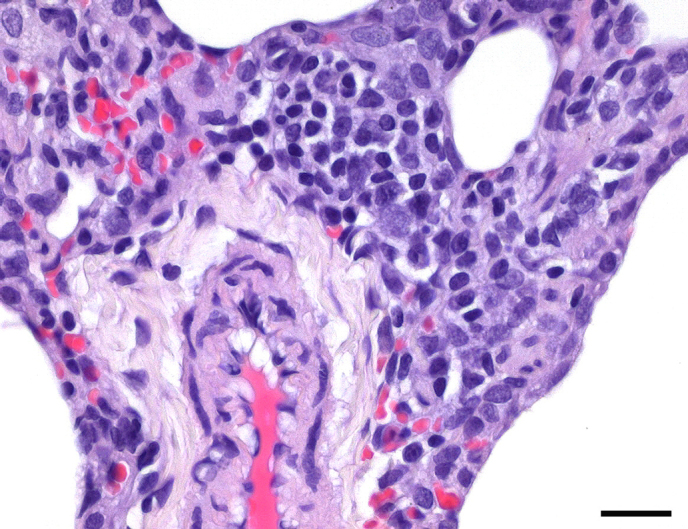
Perivascular inflammatory cell focus in an adult male common marmoset. Plasma cells, lymphocytes, and macrophages are the predominant cell type in this lesion. HE, scale bar = 20 µm.

**Figure 6:**
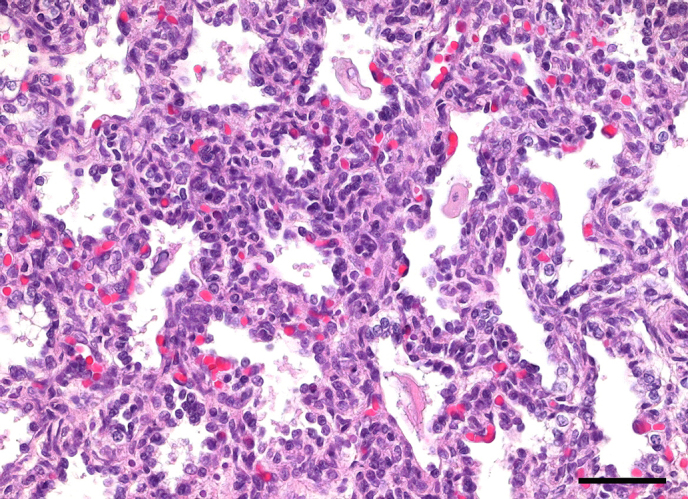
Lung immaturity with cuboidal alveolar epithelium in a female newborn common marmoset. There is intra-alveolar evidence of amniotic fluid aspiration. HE, scale bar = 50 µm.

**Figure 7:**
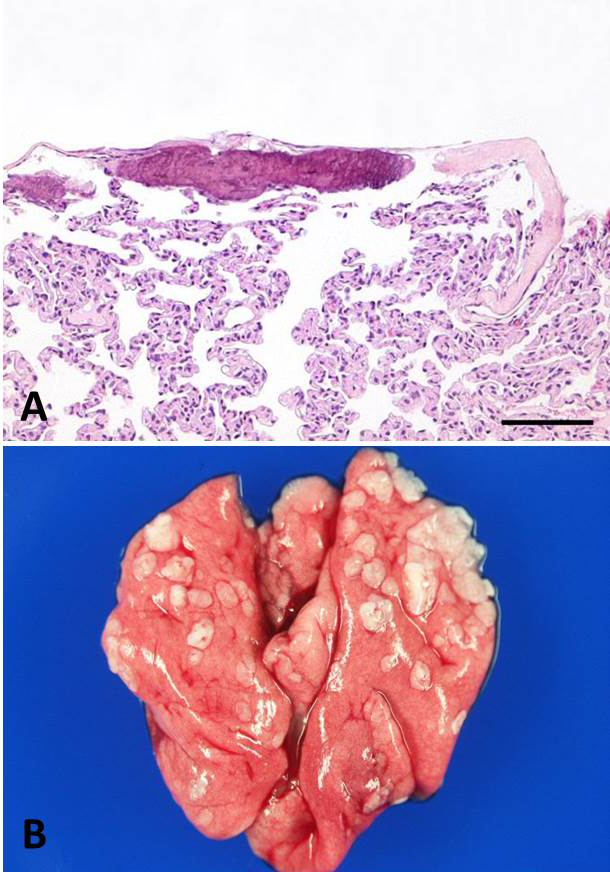
**(a, b)** Focal subpleural fibrosis with mineralization in an adult female common marmoset. HE, scale bar = 100 µm **(a)**. The corresponding macroscopic picture is characterized by multifocal subpleural plaque formation **(b)**.

**Figure 8:**
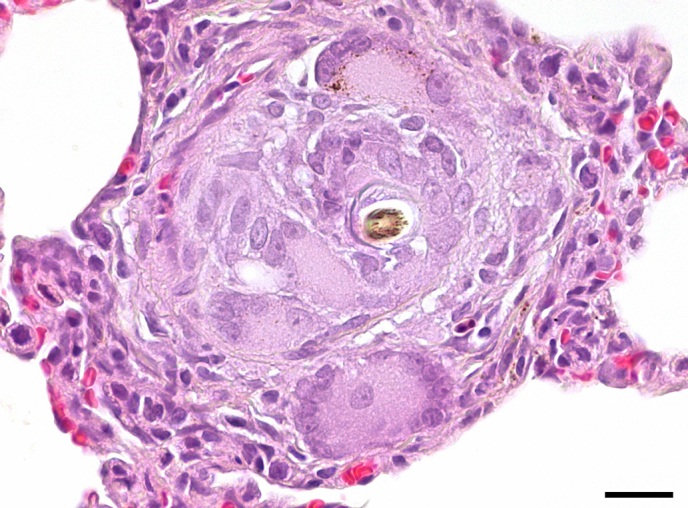
Foreign-body granuloma in an adult female common marmoset. A hair fragment is surrounded by multinucleated giant cells and macrophages. HE, scale bar = 20 µm.

Within the group of miscellaneous lung findings, extramedullary hematopoiesis, mainly characterized by megakaryocytes within alveolar septa, was observed in 74 animals (11.6 %). This predominantly affected adult marmosets, which regularly showed concurrent foci of extramedullary hematopoiesis in multiple organs (liver, spleen, etc.). The second-most-common miscellaneous finding was multifocal interstitial or subpleural mineralization, being present in 70 juvenile and adult marmosets (10.97 %), followed by disseminated inflammatory cell foci observed in 66 marmosets (10.34 %). They mainly consisted of plasma cells, macrophages, and lymphocytes and were primarily located within alveolar septa, perivascular or peribronchial/peribronchiolar (Fig. 5). Cuboidal alveolar epithelium and thick fibrotic interalveolar tissue of variable extent, indicative of pulmonary immaturity, were present in 62 newborn/stillborn marmosets and in 1 juvenile animal (9.72 %). In many cases, premature lungs also showed total atelectasis and represented a common cause of death in newborn marmosets. A few animals with immature lungs also revealed accumulations of intra-alveolar amniotic fluid (Fig. 6). Focal or multifocal alveolar histiocytosis, found in 26 marmosets (4.08 %), was generally associated with inflammatory lung lesions. Multifocal interstitial and subpleural fibrosis was detected in juvenile and adult marmosets (3.61 %), occasionally accompanied by focal mineralization (Fig. 7). Three adult marmosets (0.47 %) revealed pulmonary arteriopathy, characterized by hyperplasia and mineralization of the tunica media as well as edema and hypertrophy of the tunica intima. A focal foreign-body granuloma due to an aspirated hair fragment (Fig. 8) was observed in the lung of one adult female marmoset (0.16 %).

## 4 Discussion

With an incidence of 32.99 %, the most common inflammatory condition in the lungs was interstitial pneumonia. However, the majority of cases were mild and were not associated with severe clinical disease or death. Except for a few case reports, published data on the incidence of interstitial pneumonias in common marmoset colonies are lacking. David et al. (2009) observed pneumonias in 9 of 597 marmosets but did not provide further classification of this diagnosis. The etiopathogenesis of interstitial pneumonia generally includes aerogenous damage to the alveolar epithelium (e.g., by toxic gases or due to infection with pneumotropic viruses) or hematogenous injury to the alveolar capillary endothelium or basement membrane (e.g., in septicemia, by endotoxins from the alimentary tract, from free radicals released in acute respiratory distress syndrome, from microembolism or disseminated intravascular coagulation, in the context of hypersensitivity reactions, or due to infection with endotheliotropic viruses) (López, 2007). In the common marmosets affected by interstitial pneumonia, testing for respiratory viruses was not performed routinely. Therefore, a viral etiology accounting for at least a part of interstitial pneumonias cannot finally be excluded. Evidence of bacterial agents was present in only a few cases, including *Streptococcus* sp., *Escherichia* coli, *Klebsiella pneumoniae*, *Pseudomonas aeruginosa*, and *Erysipelothrix rhusiopathiae*. These isolates are of variable pathogenicity regarding respiratory infections but are usually not associated with interstitial pneumonia (Simmons and Gibson, 2012). In some marmosets, bacterial isolates were also obtained from other organs (gall bladder, intestine) with evidence of bacteria-induced pathologic lesions suggesting septicemic distribution of the respective bacteria. Mycotic interstitial pneumonia was observed in one marmoset with systemic candidiasis, which represents the most frequently occurring mycotic disease in immunocompromised non-human primates (Simmons and Gibson, 2012). However, the etiology of the majority of interstitial pneumonias remains unclear. Environmental influences linked to the housing conditions of the marmoset colony – e.g., room temperature, humidity, air exchange rate and air filter specifications, and aerosol formation – may represent predisposing or triggering factors for the development of interstitial lung inflammation in captive marmosets, although evidence for this assumption is lacking. In addition, fine dust pollution has to be considered as an initiating factor for interstitial lung disease, especially with regard to the atmospheric composition in the natural habitat of common marmosets, which surely differs from the artificial conditions in indoor marmoset husbandry. The presence of anthracosis in 52 marmosets (8.15 %) points to at least partial exposure of indoor-kept marmosets to the outside air. However, the association between anthracosis and interstitial pneumonia remains questionable as many animals with intrapulmonary coal dust pigment did not exhibit obvious interstitial inflammation or fibrosis, which is consistent with observations in cynomolgus monkeys (Sato et al., 2012). Finally, influences like stress or immunological status of the animal, both conditions that are hard to grasp, may have an impact on the individual's disposition to develop interstitial lung inflammation (López, 2007).

Lobar pneumonias and bronchopneumonias occurred in a small number of marmosets (1.41 and 0.94 %, respectively), were grossly evident in most cases, and were generally caused by bacterial infection resulting in severe disease and death. *Bordetella bronchiseptica* was isolated from the lungs in all cases of bronchopneumonia and 1 case of lobar pneumonia. Outbreaks of bordetellosis with characteristic pneumonic lesions have previously been described in marmoset colonies and were associated with high morbidity and mortality (Baskerville et al., 1983; Chalmers et al., 1983). Pathogenic *Klebsiella pneumoniae* strains are known to cause purulent/fibrinopurulent pneumonias in New World monkeys (Berendt et al., 1978; Simmons and Gibson, 2012) and could be isolated in most cases of lobar pneumonia in the common marmosets.

Primary pulmonary neoplasia is rare in non-human primates and is limited to a few case reports primarily referring to malignant epithelial tumors observed in different macaque species (Lowenstine and Osborn, 2012). In a previous study on the incidence of pulmonary tumors in the marmoset colony of the German Primate Center, Brack et al. (1996) reported three cases of primary lung tumors (one small-cell carcinoma, one bronchial adenoma, one squamous cell carcinoma) in 409 adult callitrichids that were examined between 1978 and 1994. However, in the present study, for which data were obtained from the time period between 1997 and 2011, pulmonary neoplasms in the marmoset colony (0.79 %) exclusively represented secondary tumors in the context of metastatic disease with primary tumors located in other organ systems (lymphatic system, mammary gland). Primary lung malignancies or benign lung tumors were not observed in the present study.

Acquired atelectasis with patchy distribution was commonly diagnosed in the examined marmoset lungs (27.9 %). However, in most cases, obvious causative factors, e.g. compression or obstruction, could not be observed. Therefore, a large portion of atelectasis probably resulted from artificial lung collapse during necropsy followed by immersion fixation with formaldehyde. Congenital atelectasis mainly affected stillborn marmosets or newborn animals that died within a couple of days after birth (5.8 %). The main causes of congenital atelectasis include obstruction of airways due to aspiration of amniotic fluid and alterations in the quantity and quality of pulmonary surfactant (López, 2007). In the common marmosets, atelectatic lungs regularly showed concurrent lung immaturity, suggesting surfactant deficiency. In a few cases, lung immaturity was associated with hyaline membrane formation, indicating acute respiratory distress syndrome as the likely cause of death. Amniotic fluid aspiration was evident in a couple of newborn marmosets and might have caused atelectasis due to airway obstruction.

Alveolar emphysema is a common secondary finding in lungs affected by bronchopneumonia or lobar pneumonia and can be attributed to a valve effect elicited by exudate plugs in the intrapulmonary airways (López, 2007). However, as the incidence of alveolar emphysema clearly exceeds the number of alveolar pneumonias in the marmoset study cohort, alveolar emphysema in most marmosets likely represents an agonal change or a euthanasia artifact. The same probably applies to most marmoset lungs exhibiting circulatory disturbances, including acute congestion, edema, and hemorrhage, which are frequently seen in animals euthanized with barbiturates (López, 2007).

Hemosiderosis is a common finding in many New World monkey species, including common marmosets; mainly manifests in the liver; and is believed to be caused by high-iron diets (Miller et al., 1997; Rensing and Oerke, 2005). As there were no signs of chronic heart failure in cases of pulmonary hemosiderosis but there was evidence of concurrent hemosiderosis in other organs, the majority of pulmonary hemosiderin deposition in the common marmosets of this study is regarded as the result of systemic iron overload due to excessive intestinal iron uptake. However, the presence of siderophages may also, to some extent, represent residua of localized pulmonary hemorrhages of undefined origin (Sato et al., 2012).

The occurrence of extramedullary hematopoiesis in the lungs of adult common marmosets has previously been described by Kaspareit et al. (2006) and is believed to be an incidental finding without clinical relevance (Zühlke and Weinbauer, 2003; Chamanza et al., 2006). Subpleural mineralization macroscopically presented as subpleural plaques, which are distinctly visible at necropsy. Both interstitial and subpleural mineralization was found in 70 marmosets (10.97 %) and was largely regarded to be of metastatic origin as there was co-existing mineralization of other tissues with accentuation on basal lamina structures. Taking into consideration that the diet for young marmosets in the German Primate Center is supplemented with vitamin D to prevent rachitic lesions, soft tissue mineralization in the common marmosets was likely due to hypervitaminosis D, which is a well-known nutritional disease entity in New World monkeys (Hunt, 1969; Kaspareit et al., 2006; McInnes, 2012; Saravanan et al., 2015). Circumscribed areas of interstitial and subpleural fibrosis occurred in 23 juvenile and adult marmosets (3.61 %) and presumably represent residua from earlier tissue damage. Mononuclear inflammatory cell foci, which were present in 66 marmoset lungs (10.34 %), are a regularly observed background finding in common marmosets and may affect different organ systems (Chamanza et al., 2006; Kaspareit et al., 2006). In the lungs, it is important to distinguish between such inflammatory cell foci and interstitial pneumonia, which should be feasible regarding the extent, distribution, and severity of infiltrating inflammatory cells. The histological features of pulmonary arteriopathy observed in 3 adult marmosets (0.47 %) were indicative of pulmonary hypertension, and concurrent hypertrophic cardiomyopathy was present in at least one of the affected animals. However, the exact pathogenetic mechanisms leading to pulmonary arteriopathy remained obscure in the common marmosets. Occasional occurrence of foreign-body granulomas in marmoset lungs has previously been reported by Kaspareit et al. (2006). They are usually caused by aspiration of foreign material (hair, food particles, plant fragments) (Sato et al., 2012). When small and focal as in the present case, they can be regarded as incidental microscopic findings without clinical relevance. However, aspiration of larger or sharp-edged foreign bodies may result in substantial tissue reaction and respiratory distress. Tissue migration may lead to complications like abscess formation, pneumo- or pyothorax, or signs of penetration of other organs (López, 2007).

This study documents the range and incidence of spontaneous histological findings in the lungs likely to be encountered in purpose-bred common marmosets used in toxicological or experimental studies. When interpreting these findings in marmoset studies, special care should be taken to identify preexisting pulmonary disease and to distinguish species-specific background findings from trial-related changes.

## 5 Data availability

All relevant data are presented in the paper. Please contact the corresponding author for further details.
